# ConEVA: a toolbox for comprehensive assessment of protein contacts

**DOI:** 10.1186/s12859-016-1404-z

**Published:** 2016-12-07

**Authors:** Badri Adhikari, Jackson Nowotny, Debswapna Bhattacharya, Jie Hou, Jianlin Cheng

**Affiliations:** 1 Department of Computer Science, University of Missouri, Columbia, MO 65211 USA; 2 Informatics Institute, University of Missouri, Columbia, MO 65211 USA; 3 C. Bond Life Science Center, University of Missouri, Columbia, MO 65211 USA

**Keywords:** Protein contact assessment, Chord diagrams, Contact maps, Contact visualization, Jaccard similarity

## Abstract

**Background:**

In recent years, successful contact prediction methods and contact-guided ab initio protein structure prediction methods have highlighted the importance of incorporating contact information into protein structure prediction methods. It is also observed that for almost all globular proteins, the quality of contact prediction dictates the accuracy of structure prediction. Hence, like many existing evaluation measures for evaluating 3D protein models, various measures are currently used to evaluate predicted contacts, with the most popular ones being precision, coverage and distance distribution score (X_d_).

**Results:**

We have built a web application and a downloadable tool, ConEVA, for comprehensive assessment and detailed comparison of predicted contacts. Besides implementing existing measures for contact evaluation we have implemented new and useful methods of contact visualization using chord diagrams and comparison using Jaccard similarity computations. For a set (or sets) of predicted contacts, the web application runs even when a native structure is not available, visualizing the contact coverage and similarity between predicted contacts. We applied the tool on various contact prediction data sets and present our findings and insights we obtained from the evaluation of effective contact assessments. ConEVA is publicly available at http://cactus.rnet.missouri.edu/coneva/.

**Conclusion:**

ConEVA is useful for a range of contact related analysis and evaluations including predicted contact comparison, investigation of individual protein folding using predicted contacts, and analysis of contacts in a structure of interest.

## Background

The success of many protein residue contact prediction methods, in the recent years, has kindled a new hope to solve the long standing problem of ab initio protein structure prediction [1–6]. Consequently, contact-guided ab initio structure prediction has emerged as an important field. When accurately predicted contacts are supplied as input to structure prediction or reconstruction methods, accurate folds can be predicted consistently [1, 7–9]. In general, accurate contacts lead to accurate structural models. However, for predicting folds of sequences which do not have homologous templates (hard sequences), the optimal way of utilizing predicted contacts is still an ongoing research. For instance, experiments on true contact reconstruction have suggested that 9 Å or more distance threshold delivers best reconstruction with Cβ atom [10, 11], but the Critical Assessment of Protein Structure Prediction (CASP)’s definition of 8 Å threshold is still widely used to predict contacts [1–3, 6, 12]. Marks et al. have even demonstrated successful structure predictions using Cα atoms and 7 Å threshold for defining contacts [13]. Similarly, it is widely accepted that long-range contacts [12, 14, 15] are the most useful of the three contact types (short-, medium-, and long-range), but some structural domains introduced in CASP like T0765-D1, T0709-D1, T0711-D1, T0756-D2, T0700-D1 have very few or no long-range contacts at all. In addition, Michel et al. discuss some examples of proteins that could not be accurately reconstructed despite high accuracy of predicted contacts in their PconsFold method [16]. Using the protein 1JWQ, Vassura et al. show how some structures cannot be folded with distance thresholds below 16 Å [10]. Zhang et al. report folding 90 transmembrane proteins at 14 Å cut-off [17]. Furthermore, in these works, no common agreement is found on the optimal number of contacts (or a range) needed for accurate reconstruction.

Hence, a tool to study the relationship between contact parameters and structure types is deemed necessary. Currently, for evaluating predicted contacts, the three most widely used evaluation measures are precision, coverage and distance distribution score (X_d_) [3, 12, 14, 18–22]. In addition, other measures like ‘mean false positive error’, ‘distance in contact map’ or ‘spread’ [13], F-score and Matthews correlation coefficient (MCC) [12] are also used for a more rigorous evaluation of the predicted contacts. Osvaldo et al. [23] had published EVAcon in 2005 that could calculate some of these measures, which no longer seems accessible. On the other hand, existing tools like CMView [24] and CoeViz [25] only enable contact map visualization and multiple sequence visualization.

In this paper, we present ConEVA, a fast web application (along with a downloadable tool) for protein contact evaluation and comparison. Besides the server, we also report some of our observations obtained through the application of our tool on larger data sets. We discuss how the length of a protein can influence various evaluation measures, the minimum number of contacts to evaluate, and the range of the evaluation measure values associated with the determination of the correct fold of a protein.

## Implementation

### Datasets

Throughout this manuscript we often refer to the dataset of 150 diverse proteins with average length of 150 residues introduced by Jones et al. in the PSICOV paper [4]. This data set along with other examples, including many CASP data sets, are provided as pre-curated data sets available through the “All Examples” link in the web server homepage.

### Contact definition

Other than the places where we explicitly mention, in this work we primarily use the CASP definition of contacts, which is – a pair of residues separated by at least 6 residues are said to be in contact if their Cβ atoms (Cα in case of Glycine) are closer than 8 Å.

### Input and interface

**Fig. 1:**
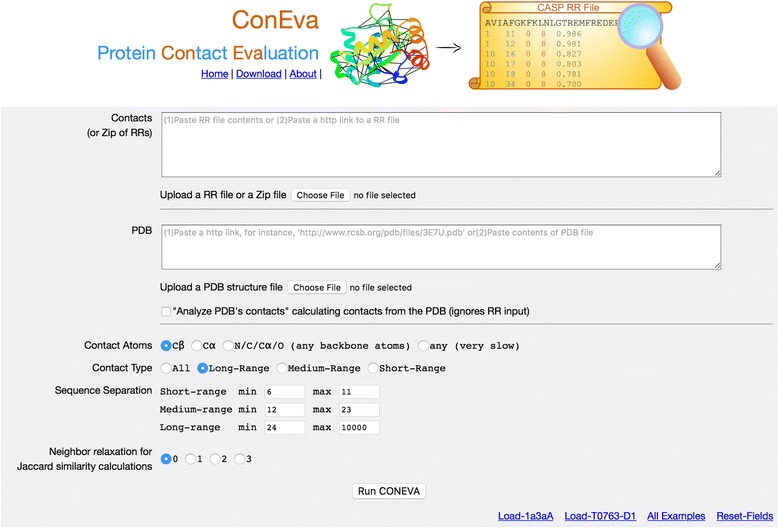
A screenshot of ConEVA homepage showing all input fields

The primary input to ConEVA is residue-residue contacts in CASP’s RR file format, whose description is available at http://predictioncenter.org/casprol/index.cgi?page=format#RR. A single RR file or multiple RR files zipped into a single zip file can be supplied. Along with predicted contacts, a native structure in PDB file format [26], may be supplied for contact evaluation. For domain based evaluations, as performed in CASP evaluations, the domain structure may be supplied as native PDB file instead of the full target structure. Besides these data inputs, the server also allows to specify if the input contacts are between Cα or Cβ atoms. In addition, a user can choose to evaluate short-, medium-, long-range, or all contacts by defining the sequence separation distances. Figure 1 shows a screenshot of ConEVA input interface. Besides allowing users to supply contact RR files, many pre-curated data sets are available through the “All Examples” link in the homepage for users to test.

### Server description

Input contacts are first sorted using the confidence column in the contact rows. Using the minimum and maximum sequence separation thresholds supplied for defining short-, medium- and long-range contacts, and the choice made for contact type (all/short-range/medium-range/long-range) contact rows that are not of a user’s interest are filtered out. If a native structure is also supplied, contact residue pairs that do not exist in the native structure are filtered out. Then, the top-5, L/10, L/5, L/2, L, and top-2 L contacts are selected and grouped for assessment. L is the length of the native chain when supplied, and otherwise, it is the length of the sequence for which contacts are predicted.

Perl and Perl CGI is used for server development, and we use ‘heatmap.2’ function in the ‘gplots’ package [27] in R for visualizing Jaccard similarity matrix, and ‘plotrix’ package [28] for drawing chord diagrams.

### Sever Output

**Fig. 2:**
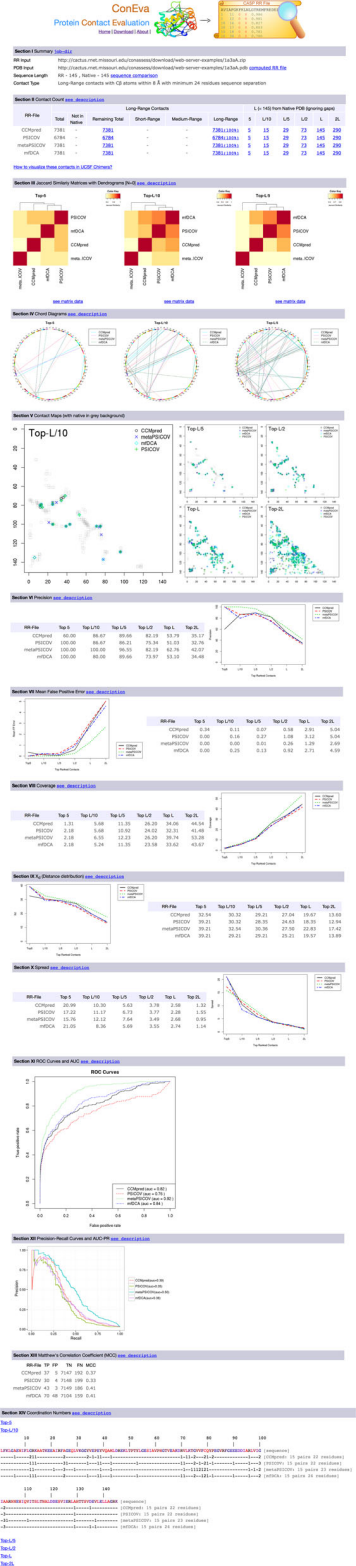
A screenshot of ConEVA output (results page) showing all result sections

The web-server output is organized in various sections. The first section summarizes the input files, contacts computed from the native structure in EVACon format [23], sequence length of contacts file and native structure with a link to the sequence comparison, and a description of the definition of contact used for all following results. The next section tabularizes contact counts for short-, medium-, and long-range contacts, and for top-5, top-L/10, etc. up to top-2 L contacts. Number of contacts that are not in native structure is also shown. In addition, if a native structure is provided as input, all numbers appear as hyperlinks to UCSF Chimera command line scripts, which can be downloaded and opened in UCSF Chimera to directly visualize the selected number of contacts within the native structure. The next section, visualizes Jaccard similarity matrices in the form of ‘heatmap’ and ‘dendrogram’ plots. The dendrogram shows similar contact sets in closer branches. Each plot has a link below it which links to the actual similarity matrix. The next section visualizes Chord diagrams. Contact maps appear in the next section, with native contact map shown in background. The subsequent sections present calculations and plots for precision, mean false positive error, coverage, X_d_, and spread. ROC curves with calculations for Area Under the Curve (AUC) are displayed next, followed by precision-recall curves. The last two sections present calculations for Matthew’s correlation coefficient and 1D visualization of coordination numbers. In the absence of a native structure, only the first five sections and the last section are reported, and further, if only a single contact prediction file is supplied, the section for Jaccard similarity calculations is skipped. A screenshot of the output is shown in Fig. 2.

### Measures computed on contacts

For each group of selected top contacts, coordination numbers [29] and contact maps are shown as 1D and 2D visualizations. Coordination number defines the number of contacts that a residue is involved in. Realizing the importance of contact assessment in the absence of a native structure, we introduce visualization and comparison using chord diagrams. See Discussion section for illustrations.

### Quality measures with respect to native structure

For each group of these selected contacts the following evaluation measures are calculated: precision, coverage, mean false positive error, distance distribution score (X_d_) [3, 12, 14, 18–22], Spread [13], MCC [12], AUC_PR [30]. Precision is defined as the percentage of correctly predicted contacts, calculated as the ratio of the number of predicted contacts that are correct and the number of predicted contacts selected for evaluation, $Precision = \frac{TP}{TP+FP}$. The true positives (TP) and false positives (FP) are the number of correctly and incorrectly predicted contacts. For instance, when we select top five contacts for evaluation, TP + FP is fixed at five and TP can range from 0 to 5. Coverage is the percentage of true contacts contained in a predicted list of contacts, calculated as the ratio of the number of correctly predicted contacts and the total number of contacts in the native structure, $Coverage=\frac{TP}{N_c}$, where ${N}_c$ is the number of true contacts in the native structure. Mean false positive error is calculated as the mean of absolute deviation of all the incorrectly predicted contacts, $Mean\ FP\  Error = \frac{1}{FP}{\displaystyle \sum}\left({d}_{ij}-d\right)$, where d is the distance threshold for the contact definition (usually 8 Å) and d_ij_ is the actual distance of a false positive pair of predicted contacts in the native structure.

The distance distribution score (X_d_) measures the weighted harmonic average difference between the predicted contacts distance distribution and the all-pairs distance distribution. While predicted contact distance distribution refers to the distribution of actual distances for the predicted contacts, all-pairs distance distribution is the distribution of distances for all the true contacts in the native structure. X_d_ is calculated as,

$$ {X}_d = {\displaystyle {\sum}_{i=1}^{15}}\frac{PiP-PiA}{d_i*15} $$

where the sum runs for 15 distance bins covering the range from 0 to 60 Å. *d*_*i*_ is the distance representing each bin, its upper limit (normalized to 60). *PiP* is the percentage of predicted pairs whose distance is included in bin *i. PiA* is the same for all the pairs and is zero for all bins with *d*_*i*_ > 8 Å, such that the value of X_d_ increases heavily because of the contacts that are very incorrect, i.e. the contacts whose true distance is very large. Defined in this way, although the harmonic average reflects the difference between the real and predicted distances of residues, interpreting the meaning of a particular valued of X_d_ can be difficult. In general, for a given set of predicted contacts, X_d_ > 0 indicates the positive cases where at least some contacts in the set are correct, whereas when X_d_ is closer to 0, the set can be considered random contacts.

Spread [13] is computed using contact maps. For a given set of predicted contacts, it is the mean of the distances from every true contact to the nearest predicted contact in 2D contact map.

$$ Spread=\frac{1}{N_c}{\displaystyle \sum_{i=1}^{Nc}} \min \left\{ dist\left({T}_i-P\right)\right\} $$

where N_c_ is the number of true contacts, T_i_ is a true contact in the native structure, and ***min***{***dist***(***T***_***i***_ − ***P***)} is the minimum Euclidean distance between the true pair T_i_ and all predicted residue pairs in the 2D contact map where every residue sequence separation is considered a unit.

### Measures of similarity between predicted sets

In addition, for computing similarity between predicted contacts in the absence of native structure we introduce Jaccard similarity matrix [31] computations with neighborhood relaxation. For each pair of input contact sets, say A and B, we compute the Jaccard similarity score between A and B, ***J***_***AB***_ as ${J}_{AB}=\frac{\left|A{\displaystyle \cap }B\right|}{\left|A{\displaystyle \cup }B\right|}$ where |A∩B| is the number of common contacts (intersection) between sets A and B, and |A⋃B| is the count of contacts in the set A union B. This similarity computation can evaluate to very small percentages in case of hard predictions because two sets must have precisely the same residue pair to be common, especially when we are evaluating top five or top L/10 contacts. For this reason, we introduce the idea of relaxing the similarity computation by considering contacts with ± N residue number deviation as same contact (N may be selected as 0, 1, 2 or 3). For instance, if set A has a pair 3–15 and set B has a pair 3–16, they may be considered as the same contact at N equal to 1. However, high similarity observed with N more than 1 in helical proteins can be sometimes misleading because shifts of two or more residues can have dramatic effect on the quality of the models generated using the contacts.

Besides these “reduced list” metrics [32] that only evaluate selected top contacts, ConEVA also presents “full list” metrics including Matthew’s correlation coefficient (MCC), area under the precision-recall curve (AUC_PR) [30], and Receiver Operating Characteristic (ROC) curve. To calculate MCC for a set of predicted contacts, all contacts having confidence more than 0.5 are considered as predicted contacts to calculate true positive (TP), true negative (TN), false positive (FP) and false negative (FN) so that

$$ MCC=\frac{TP\ast TN-FP\ast FN}{\sqrt{\left(TP+FP\right)\left(TP+FN\right)\left(TN+FP\right)\left(TN+FN\right)}} $$

### Contact prediction and model generation

Throughout this work, we use the publicly available contacts predicted by PSICOV [4]. In addition, we also installed a local copy of the tools coevolution based tool CCMpred [2], pure machine-learning based method DNcon [3], and a hybrid method MetaPSICOV [1] to make contact predictions for various data sets including the PSICOV data set of 150 proteins. These contacts along with secondary structures predicted using PSIPRED [33] were used for building models using CONFOLD [8], a fragment-free ab initio method that we recently developed to build 3D models from scratch. As discussed in the CONFOLD paper, for each protein, we selected various top predicted contacts (top-5, L/10, L/5, L/2, L, and 2 L) and built models using subsets, resulting in a total of 400 models for each protein. We selected the best model out of 400 for our analysis.

To study how various evaluation measure correlate to the final quality of models reconstructed using the predicted contacts, we build 3D models with CONFOLD using the contacts predicted for the 150 proteins in the PSICOV dataset. We argue that the TM-score [34] of the best model can be used as a score that suggests the best utility of the predicted contacts.

## Results

### Dependence of evaluation measures on L

The length of the sequence may be ignored when we are evaluating and comparing contacts predicted for a single protein sequence. However, when we are comparing contact prediction methods on more than one protein sequence and the sequences are not of same length, sequence length can bias the comparisons. For instance, if the evaluation measures we choose to make the comparison is influenced by the length of the sequence and penalizes longer sequences more, then the methods that perform poorly particularly on longer sequences can be ranked lower than they should. This is also the reason why evaluation measures like TM-score were introduced to address the limitations of measures like RMSD. Thus, it is important to study how various contact evaluation measures are correlated to the length of the protein sequence.

To study the relationship between length of the protein (L) and the quality of contacts suggested by the various contact evaluation measures, we computed Spearman’s rank correlation coefficient between the length of the protein and the evaluation measures – precision, coverage, X_d_, mean false positive error, and spread – for the long-range contacts (with sequence separation more than 23) predicted in the PSICOV dataset. In Table 1 we show that mean false positive error is the measure most uncorrelated with the length of a protein, followed by precision values for all contact selections (top-5 to top-2 L). Spread and coverage are more correlated with the length at lesser contact selections (top-5, top-L/10 and top-L/5) whereas X_d_ is more correlated with L when we select more contacts for evaluation (top-L/2, top-L, and top-2 L). Similar correlation values were obtained for the number of contacts in a protein (N_c_). In summary, these observations lead us to argue that precision and mean false positive error are the most reliable measures when comparing contact predictions.

**Table 1:** Spearman’s rank correlation coefficient between the length of a protein (L) and evaluation measures for PSICOV predicted long-range contacts in the PSICOV data set. It shows that spread, coverage and X_d_ are more correlated to L and N_c_ than precision and mean false positive error, especially below top-L contact selection. For this dataset, the lengths are distributed in the range [50, 266] with mean and standard deviation of 145 and 52 respectively

Contact-Selection	Top-5	Top-L/10	Top-L/5	Top-L/2	Top-L	Top-2 L
L vs Precision	−0.01	−0.07	0.06	0.24	0.26	0.27
L vs Coverage	−0.88	−0.59	−0.51	−0.34	−0.30	−0.31
L vs X_d_	0.31	0.35	0.46	0.49	0.51	0.55
L vs FP-Error	−0.05	0.04	−0.06	−0.16	−0.01	0.21
L vs Spread	0.88	0.66	0.60	0.58	0.55	0.57

### Number of contacts to evaluate

How many contacts should we evaluate, top-5 or top-L or top-2 L? On one hand, reconstruction studies using true contacts focus on the minimum number of contacts needed to recover the fold of a protein. For instance, DE et al. suggest that 1 contact in every 12 residues is sufficient to robustly fold a protein at topology level [35]. This translates to L/12 predicted contacts if we assume that the contacts are spread out without any overlaps. In a similar study, introducing a novel cone-peeling algorithm, Sathyapriya et al. suggest that as little as 8% of the native contacts are sufficient to determine the tertiary structure [36]. On the other hand, contacts are currently evaluated on a wide range of contact selections. It is a common practice for CASP assessors to evaluate top-5, top-L/10, and top-L/5 predicted long-range contacts. Similarly, recent contact prediction methods that utilize the predicted contacts to build three dimensional models discuss evaluating top-L/10, L/5, L/2, up to top-L contacts [2, 4, 6].

**Fig. 3:**
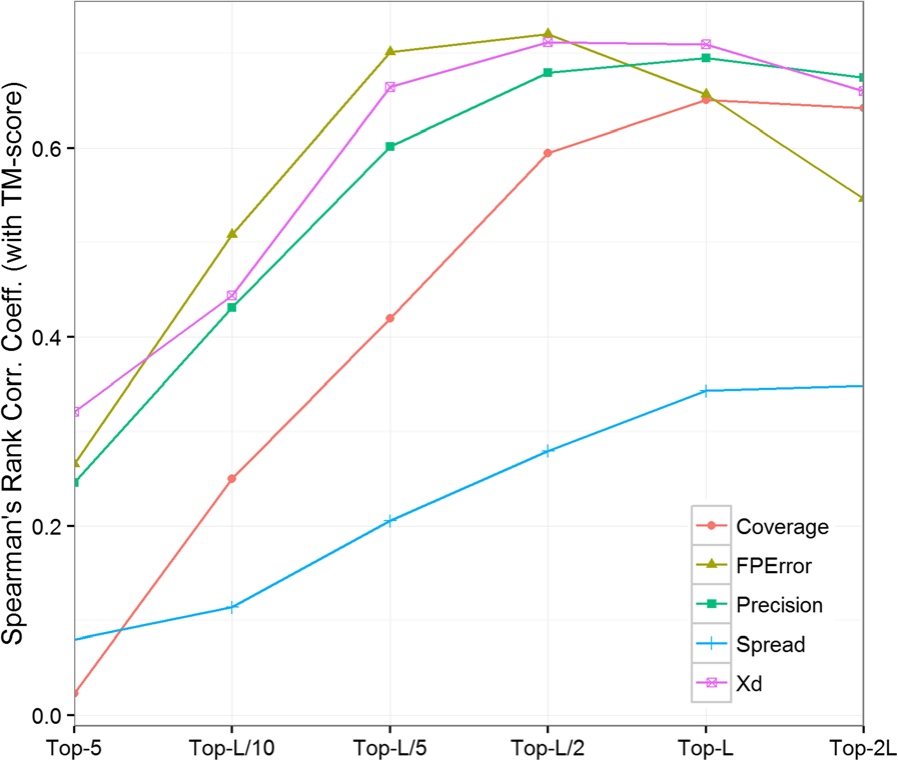
Spearman’s rank correlation coefficient between the evaluation measures (coverage, mean false positive error, precision, spread, and X_d_) and TM-score of the reconstructed models against various contact selections (top-5, top-L/10, etc.), for long-range contacts in the 150 proteins in PSICOV data set. The correlation values for mean false positive error and spread are negated to show all measures in the same quadrant

We argue that the minimum set of contacts for which there is a high correlation between the quality of contacts and the quality of the reconstructed models, is the optimal number of contacts we can evaluate. To test this, in the PSICOV data set, we calculated the Spearman’s rank correlation coefficients between the evaluation measures (precision, coverage, X_d_, spread, and mean false positive error) and the TM-score of the best CONFOLD reconstructed model, for various contact selections. The plot of correlation against top contact selections in Fig. 3, shows that correlation for the three important measures precision, X_d_, and mean false positive error, is high for at least top-L/5 contacts. In summary, we find that top-L/5 is the minimum number of long-range contacts to evaluate.

### Expected TM-score for values of evaluation measures

**Fig. 4:**
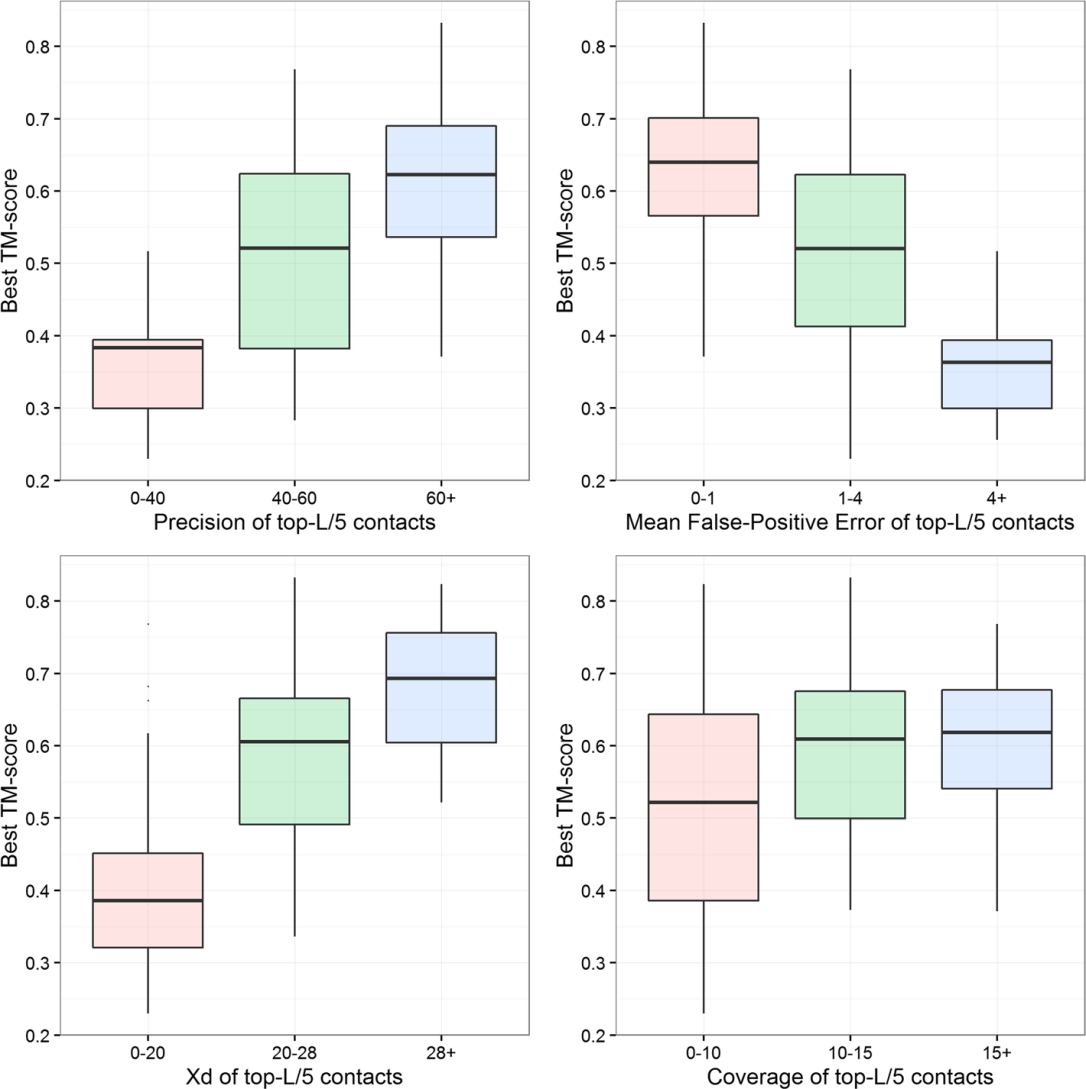
Expected TM-score of the best model reconstructed using CONFOLD against precision, mean false positive error, X_d_, and coverage bins. Top-L/5 contacts predicted by PSICOV for the 150 proteins in the PSICOV data set were used as input for the calculations

For a given protein, what values of precision, coverage, X_d_, or mean false positive error of predicted contacts may fold the protein accurately (with TM-score > 0.5)? For the contacts predicted using PSICOV [4] for the 150 proteins in the PSICOV data set we classified top-L/5 long-range contacts into 3 bins for each measure. We binned predicted contacts into three precision bins – 0 to 40%, 40% to 60% and 60+ %, three X_d_ bins – 0 to 20, 20 to 28, and 28+, three mean false positive error bins – 0 to 1, 1 to 4, and 4+, and three coverage bins – 0–10, 10–15, 15+, and observed the distribution of TM-score values in each bins. The thresholds for these bins were selected by clustering the TM-scores into three clusters. We find that on average at least 40-60% precision is required to get a TM-score of 0.5 when folding using predicted contacts only; see Fig. 4. We also find that to get similar TM-score, X_d_ should be more than 20, mean false positive error should be less than 4 and coverage should be more than 10. It is important to also note that coverage and X_d_ are also dependent upon the length of the protein unlike precision and mean false positive error.

### Protein types and evaluation measures

**Fig. 5:**
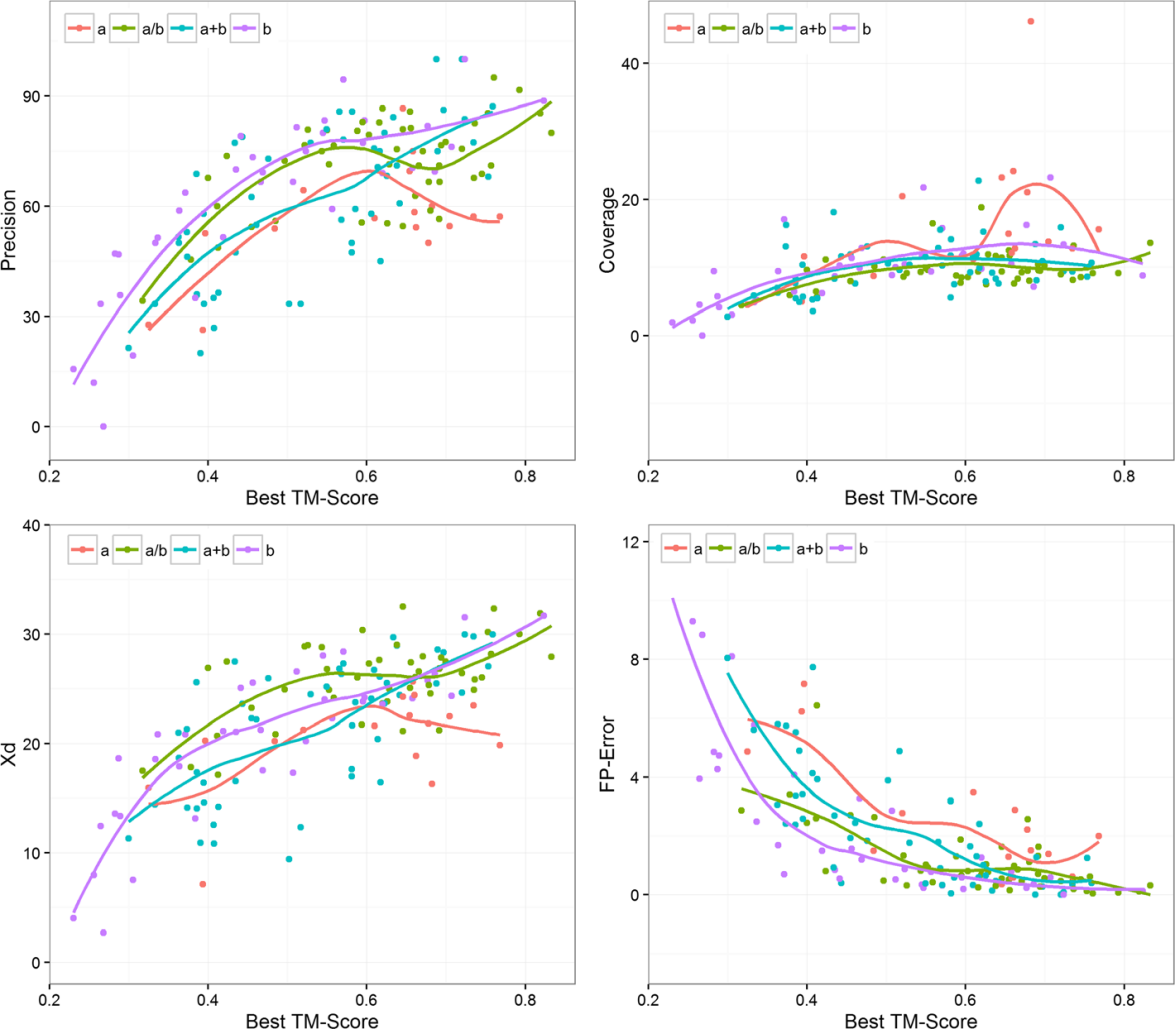
Relationship between precision, coverage, mean false positive error, and X_d_ with the best TM-score for various protein folds. It shows that β proteins are best evaluated using precision and X_d_ and coverage is relatively most important for α proteins. Evaluations are performed on top L/5 long-range contacts predicted by PSICOV and TM-score is that of the best model built using CONFOLD

Using ConEVA we studied how the evaluation of predicted long-range contacts vary for the various protein folds (α, α + β, α/β, β) in the PSICOV data set. We find that mean false positive error has the highest correlation with the TM–score of the models for all protein folds, except for β proteins. For α proteins, mean false positive error and spread have the highest correlation with TM-score suggesting that α proteins are better evaluated using these two measures than others. For α + β and α/β proteins we observed that coverage has much lower correlation than other measures (X_d_, precision, and mean-false-positive-error). All correlations are presented in Table 2 and visualized in Fig. 5. Similar statistics were observed when we selected “all” contacts instead of long-range.

**Table 2:** Spearman’s rank correlation coefficient calculations of L, N_c_, and various evaluation measures with TM-score of the best CONFOLD built model for various protein fold types. Top-L/5 PSICOV predicted contacts are evaluated

	α	α + β	α/β	β
L	−0.34	0.21	0.05	−0.13
N_c_	−0.47	0.27	0.08	−0.14
Precision	0.33	0.67	0.38	0.85
Coverage	0.60	0.33	0.28	0.70
X_d_	0.31	0.69	0.44	0.84
Mean false positive error	−0.48	−0.78	−0.63	−0.86
Spread	−0.48	0.02	−0.30	−0.29

### Similarity between predicted contacts

**Fig. 6:**
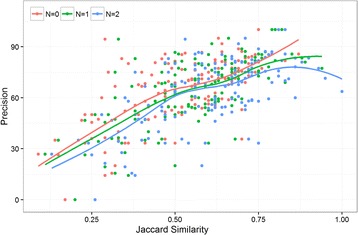
Precision of top-L/5 PSICOV predicted contacts versus the Jaccard similarity score between PSICOV contacts and CCMpred predicted contacts for the 150 proteins in PSICOV data set. N corresponds to the neighborhood size in computing Jaccard similarity

No methods currently exist for assessing the quality of predicted contacts in the absence of native structures. Since Jaccard similarity score provides a quantitative comparison of contact sets, we hypothesized that when there is larger agreement between multiple sets of predicted contacts, the confidence of the contact prediction for the protein is higher. Using the same PSICOV data set, we first computed the Jaccard similarity between the PSICOV predicted contacts and CCMpred predicted contacts, and then calculated the Spearman’s rank correlation coefficient between this similarity and the precision of the predicted contacts (see Fig. 6). High correlation coefficients of 0.63, 0.64, and 0.57 for N (neighborhood relaxation for computing Jaccard similarity) equal to 0, 1, and 2 respectively validates our hypothesis. These findings, although obvious (i.e., accurate contacts will be correlated), can have interesting applications. For instance, a very wide range of features are used for developing protein model quality assessment (QA) methods, including many contact related scores [37–39]. Jaccard similarity score is a potentially useful feature for developing QA methods. In addition, this similarity score can even be integrated into model building methods like FUSION [40], UniCon3D [41], and FRAGFOLD [42] to decide the weight of the contact energy term.

## Discussion

ConEVA allows a user to choose from various contact types, distance thresholds, and sequence separation thresholds for defining contacts and enables study of how the various measures change over various numbers of top contacts. It accepts contacts in Critical Assessment of protein Structure Prediction (CASP) RR file format. We verified ConEVA evaluations by comparing against the CASP evaluations available at http://predictioncenter.org. A downloadable version is also available that calculates all the quantitative measures without any visualizations. Below we outline some of its features with the evaluation of predicted long-range Cβ contacts for the protein ‘1aa3’ (chain A) in the PSICOV data set and protein domain T0763-D1 in the CASP11 data set as reference examples.

### Contact evaluation

**Fig. 7:**
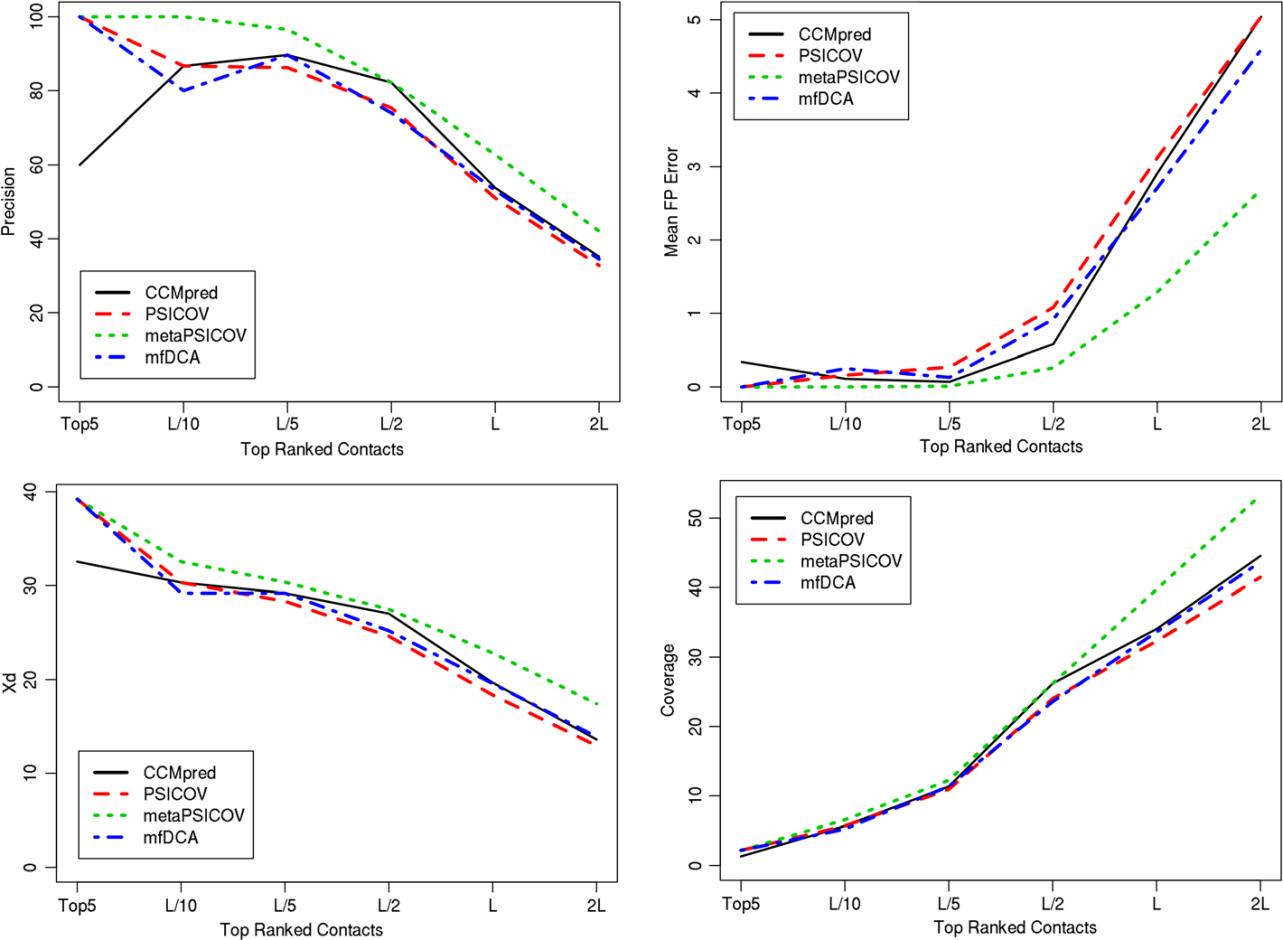
A screenshot of ConEVA evaluation of contacts predicted for the protein ‘1a3aA’ showing calculations for precision (top left), mean false positive error (top right), X_d_ (bottom left), and coverage (bottom right). For this protein, MetaPSICOV has shown slightly better performance than CCMpred, PSICOV, and mfDCA in every evaluation measure

**Fig. 8:**
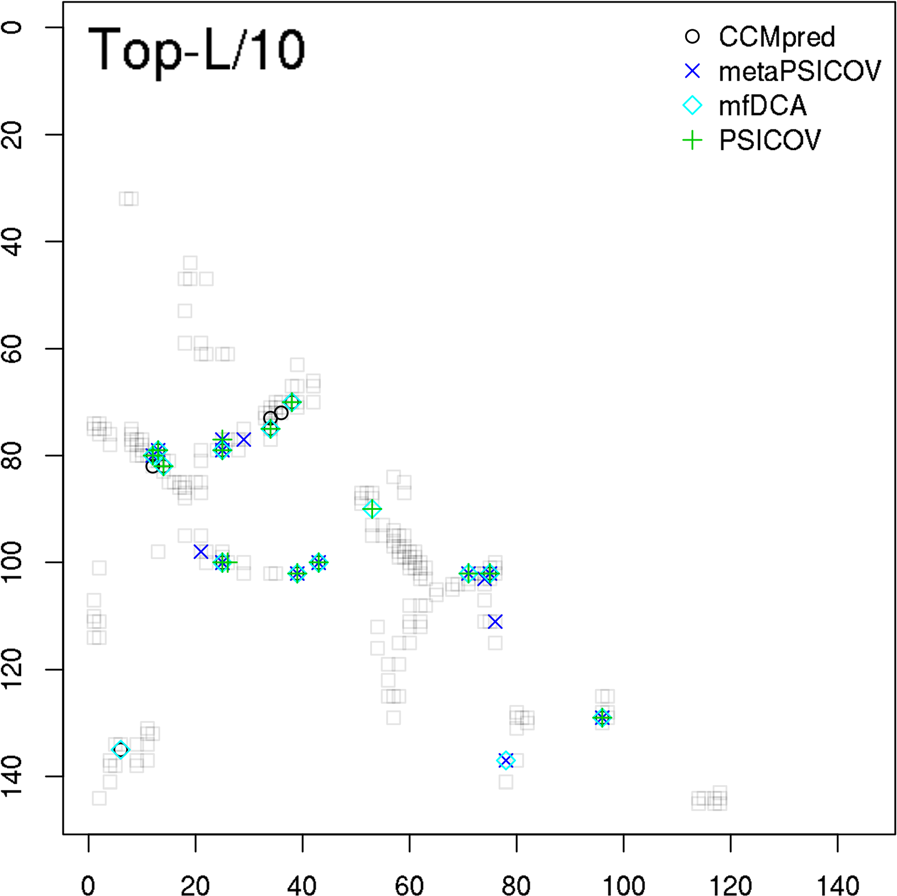
A screenshot of contact map showing long-range contacts for top-L/10 predicted contacts for the protein ‘1a3a’ with the native contacts shown in gray in background

**Fig. 9:**
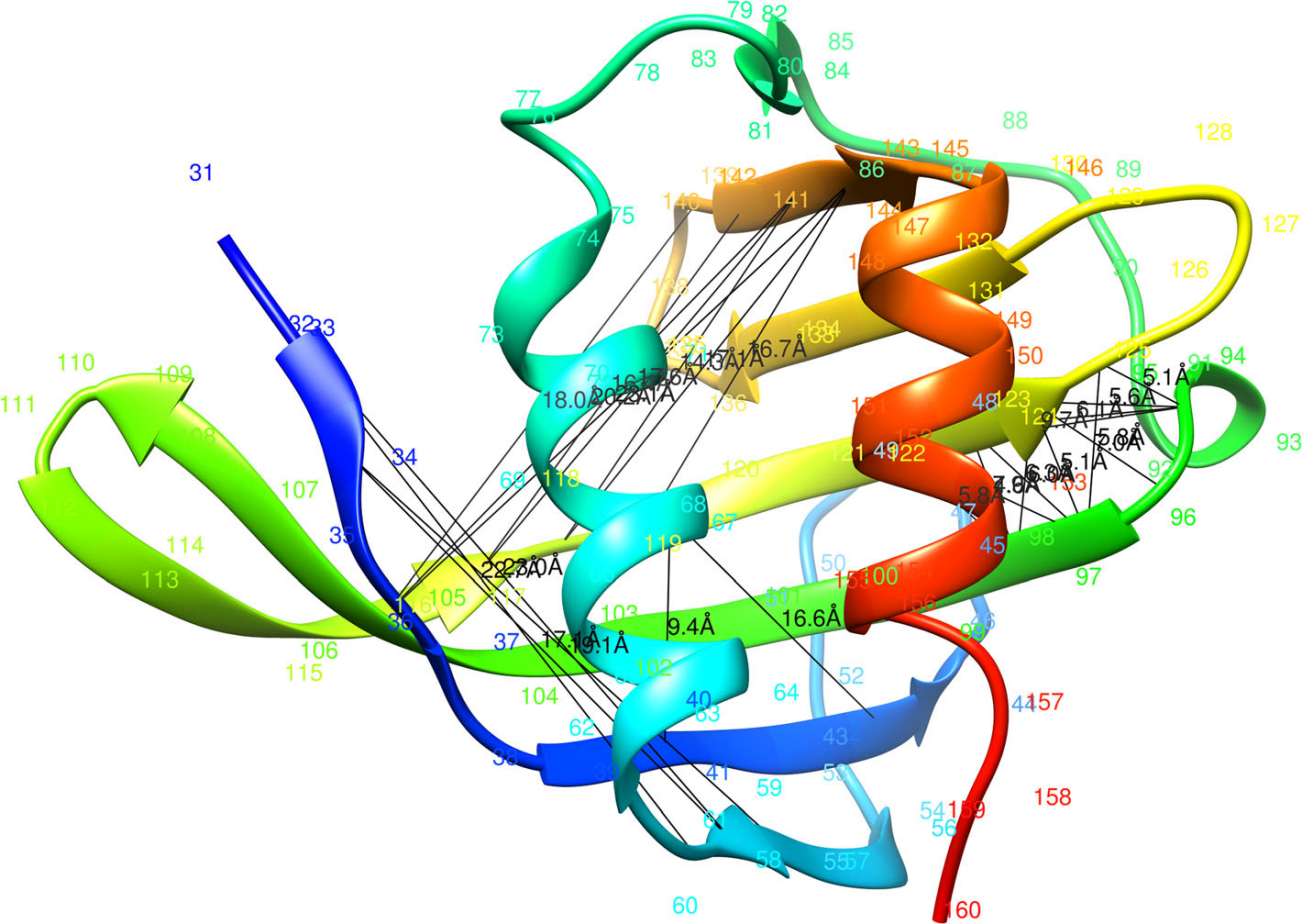
Top-L/5 CONSIP2 predicted long-range contacts (total 26 contacts) shown in the native structure domain of T0763-D1 as an example of visualizing the contacts in UCSF Chimera using ConEVA downloaded scripts. This visualization shows the clustering of the predicted CONSIP2 contacts in three regions and mostly between the beta strands, where one cluster (on the right) is correct and two other clusters are mostly wrong (with long black lines showing the distance between predicted contacts)

For predicted contacts, ConEVA evaluates the top five, L/10, L/5, L/2, L and top 2 L contacts against a native structure using precision, coverage, X_d_, mean false positive error, spread, MCC, AUC_PR, and ROC curves (see Fig. 7). For analysis and comparison, it also produces neat plots of two dimensional contact maps. For convenient comparison, in the presence of a native structure, contact maps are displayed with the native structure’s contact maps in the background (see Fig. 8). For visualizing predicted contacts in the native structure, UCSF Chimera command scripts [43] are provided to download and run locally (see Fig. 9).

### Contact assessment in the absence of a native structure

**Fig. 10:**
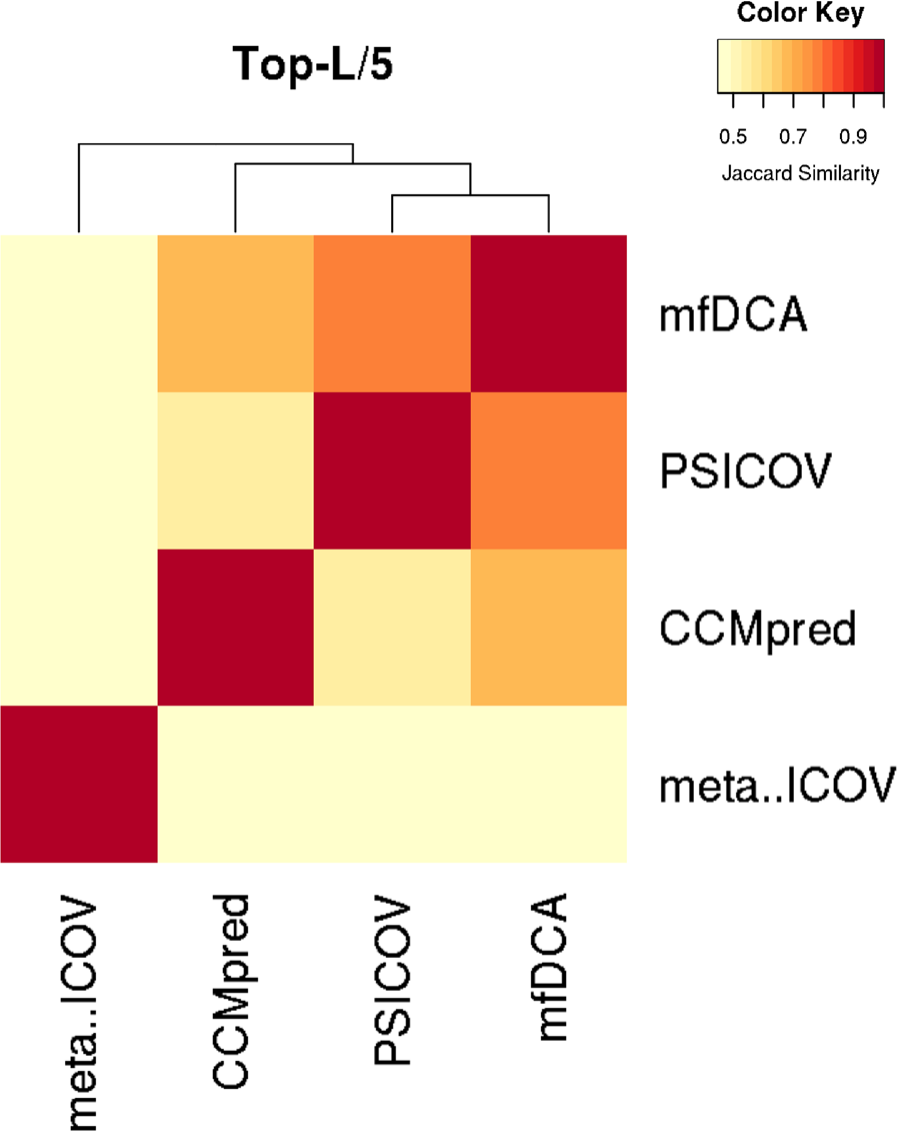
A screenshot of Jaccard similarity matrix visualization of contacts predicted for the protein 1a3a chain A. The Jaccard similarity matrix with N equals 0 (right) shows that contacts predicted by mfDCA and PSICOV are most similar and MetaPSICOV contacts are equally similar to all other predictions

**Fig. 11:**
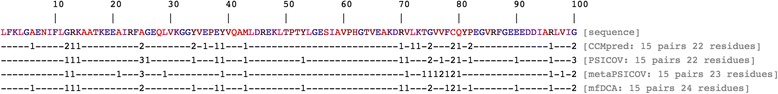
A screenshot of 1D visualization of coordination numbers within the first 100 residues of the protein ‘1aa3’. Each row represents the contacts predicted by a single method, with number of contacts and number of residues involved in the contacts shown at the end. From this visualization, three clusters of contacts can be observed as common between the four methods

**Fig. 12:**
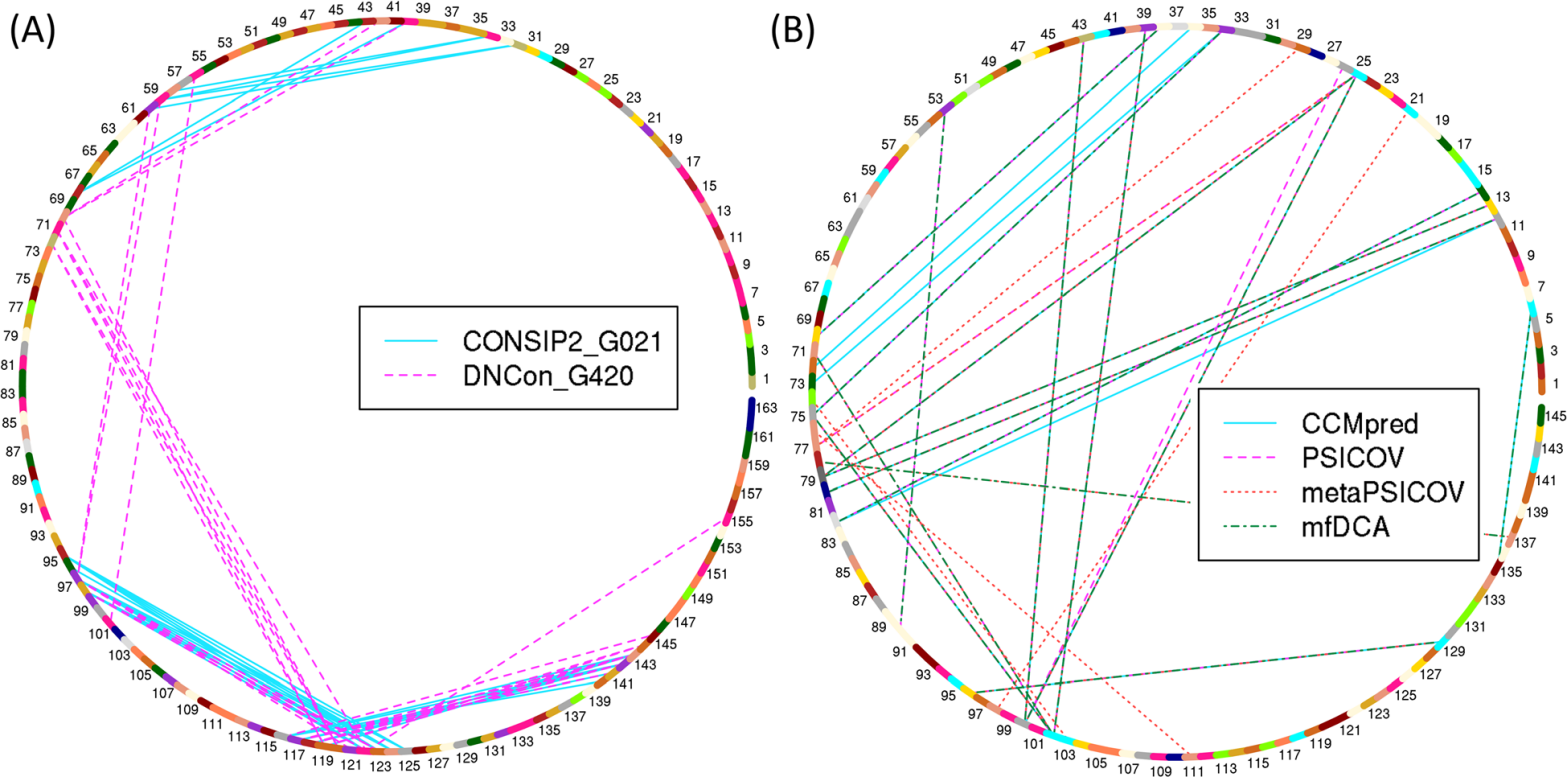
Chord diagrams for top-L/10 contacts for T0763-D1 (**a**) and for top-L/10 contacts predicted for ‘1aa3’ (**b**). The diagrams show that contacts predicted for T0763-D1 are clustered with no contacts predicted for the first 30 residues (which is in fact a disordered region with no native coordinates), whereas, predicted contacts have high overlaps between methods and are well spread for ‘1aa3’

When only predicted contacts (or multiple set of contacts) are submitted, two dimensional contact maps and one dimensional coordination numbers are presented along with counts for short-, medium-, and long-range contacts and visualizations using contact maps, chord diagrams and Jaccard similarity matrixes along with dendrograms (see Fig. 10). The visualization of coordination numbers serves as a detailed analysis of the residue location of predicted contacts (see Fig. 11). When analyzed along with predicted three-state secondary structures (helix, strand, and coil), coordination numbers can show the contrast or agreement between predicted secondary structures and contacts. For instance, clusters of predicted contacts are expected in the strand regions. Similarly, Chord diagrams can be useful to observe contact clusters, similarities in predicted contacts and even to predict disordered regions (see Fig. 12). Both, coordination numbers and Chord diagrams can also be useful to detect predicted contacts that have extremely low coverage, i.e. highly clustered contact predictions. Identifying such predictions and prediction methods can help us make decisions on using more contacts from the same source or resort to other methods of contact prediction. These results can be useful for predictive analysis of contacts to study how the contacts may be selected and/or combined for building models.

### Analysis of a structure’s contacts

A three-dimensional protein data bank (PDB) structure [26] file or a ‘pdb id’ may be provided as input to study its true contacts for a chosen definition of contacts. This feature is useful not only to study the reconstruction of a protein but also to understand the maximum and minimum values of measures like X_d_ for a structure, also allowing us to investigate what contact definitions yield a desired set of contacts for a structure of interest. This is sometimes important to investigate whether some protein structure has too few or no long range contacts at all.

## Conclusion

Contacts are becoming increasingly useful not just for ab initio protein structure prediction but also for being integrated into experimental methods, and we are finding many more applications of contacts with the increasing research on contacts. We hope that ConEVA will be useful not only to contact prediction developers but also to general public who need to predict structures for their sequences that do not have a good template.

## Abbreviations

1D: One dimensional
3D: Three dimensional
AUC_PR: Area under the precision-recall curve
CASP: Critical assessment of protein structure prediction
TP: True positives
FN: False negatives
FP: False positives
L: Length of a protein’s sequence
MCC: Matthew’s correlation coefficient
F-score: F1-score or F-measure
ML: Machine learning
N: Neighborhood size for computing Jaccard similarity score
N_c_: Number of true contacts in a protein
PDB: Protein data bank
QA: Quality Assessment
ROC: Receiver operating characteristic
TN: True negatives
X_d_: Distance distribution score

## References

[1] Jones DT, Singh T, Kosciolek T, Tetchner S (2015). MetaPSICOV: Combining coevolution methods for accurate prediction of contacts and long range hydrogen bonding in proteins. Bioinformatics.

[2] Seemayer S, Gruber M, Söding J (2014). CCMpred - Fast and precise prediction of protein residue-residue contacts from correlated mutations. Bioinformatics.

[3] Eickholt J, Cheng J (2012). Predicting protein residue-residue contacts using deep networks and boosting. Bioinformatics.

[4] Jones DT, Buchan DWA, Cozzetto D, Pontil M (2012). PSICOV: Precise structural contact prediction using sparse inverse covariance estimation on large multiple sequence alignments. Bioinformatics.

[5] Cheng J, Baldi P (2007). Improved residue contact prediction using support vector machines and a large feature set. BMC Bioinformatics.

[6] Kaján L, Hopf TA, Kalaš M, Marks DS, Rost B (2014). FreeContact: fast and free software for protein contact prediction from residue co-evolution. BMC Bioinformatics.

[7] Marks DS, Hopf TA, Sander C (2012). Protein structure prediction from sequence variation. Nat Biotechnol.

[8] Adhikari B, Bhattacharya D, Cao R, Cheng J (2015). CONFOLD: Residue-residue contact-guided ab initio protein folding. Proteins.

[9] Kosciolek T, Jones DT (2014). De novo structure prediction of globular proteins aided by sequence variation-derived contacts. PLoS One.

[10] Vassura M, Margara L, Di Lena P, Medri F, Fariselli P, Casadio R (2008). Reconstruction of 3D structures from protein contact maps. IEEE/ACM Trans Comput Biol Bioinform.

[11] Duarte JM, Sathyapriya R, Stehr H, Filippis I, Lappe M (2010). Optimal contact definition for reconstruction of contact maps. BMC Bioinformatics.

[12] Monastyrskyy B, D’Andrea D, Fidelis K, Tramontano A, Kryshtafovych A (2014). Evaluation of residue-residue contact prediction in CASP10. Proteins Struct Funct Bioinforma.

[13] Marks DS, Colwell LJ, Sheridan R, Hopf TA, Pagnani A, Zecchina R, et al. (suppl info) Protein 3D structure computed from evolutionary sequence variation. Sali A, editor. PLoS One. Public Library of Science; 2011;6:e28766. 10.1371/journal.pone.0028766 PMC3233603 22163331

[14] Monastyrskyy B, Fidelis K, Tramontano A, Kryshtafovych A (2011). Evaluation of residue-residue contact predictions in CASP9. Proteins.

[15] Cheng J, Wang Z, Tegge AN, Eickholt J (2009). Prediction of global and local quality of CASP8 models by MULTICOM series. Proteins Struct Funct Bioinforma.

[16] Michel M, Hayat S, Skwark MJ, Sander C, Marks DS, Elofsson A (2014). PconsFold: Improved contact predictions improve protein models. Bioinformatics.

[17] Zhang H, Huang Q, Bei Z, Wei Y, Floudas CA (2016). COMSAT: Residue contact prediction of transmembrane proteins based on support vector machines and mixed integer linear programming. Proteins Struct Funct Bioinforma.

[18] Di lena P, Nagata K, Baldi P (2012). Deep architectures for protein contact map prediction. Bioinformatics.

[19] Ezkurdia I, Graña O, Izarzugaza JMG, Tress ML, Ezkurdia L, Grana O (2009). Assessment of domain boundary predictions and the prediction of intramolecular contacts in CASP8. Proteins.

[20] Graña O, Baker D, MacCallum RM, Meiler J, Punta M, Rost B, Tress ML, Valencia A. CASP6 assessment of contact prediction. Proteins. 2005;61:214–24. doi:10.1002/prot.20739. 10.1002/prot.20739 16187364

[21] Izarzugaza JMG, Graña O, Tress ML, Valencia A, Clarke ND. Assessment of intramolecular contact predictions for CASP7. Proteins. 2007;69:152–58. doi:10.1002/prot.21637. 10.1002/prot.21637 17671976

[22] Tegge AN, Wang Z, Eickholt J, Cheng J (2009). NNcon: Improved protein contact map prediction using 2D-recursive neural networks. Nucleic Acids Res.

[23] Graña O, Eyrich VAA, Pazos F, Rost B, Valencia A (2005). EVAcon: A protein contact prediction evaluation service. Nucleic Acids Res.

[24] Vehlow C, Stehr H, Winkelmann M, Duarte JM, Petzold L, Dinse J (2011). CMView: Interactive contact map visualization and analysis. Bioinformatics.

[25] Baker FN, Porollo A (2016). CoeViz: a web-based tool for coevolution analysis of protein residues. BMC Bioinformatics.

[26] Berman HM (2000). The protein data bank. Nucleic Acids Res.

[27] Warnes GR, Bolker B, Bonebakker L, Gentleman R, Liaw WHA, Lumley T, et al. gplots: Various R Programming Tools for Plotting Data. R Packag. version 2.17.0. 2015;2015.

[28] Lemon J. Plotrix: a package in the red light district of R. R-News. 2006;6(4):8–12.

[29] Pollastri G, Baldi P, Fariselli P, Casadio R (2002). Prediction of coordination number and relative solvent accessibility in proteins. Proteins Struct Funct Genet.

[30] Davis J, Goadrich M. The Relationship Between Precision-Recall and ROC Curves. Proc. 23rd Int. Conf. Mach. Learn. -- ICML’06. 2006;233–40.

[31] Gilbert G (1972). Distance between Sets. Nature.

[32] Monastyrskyy B, D’Andrea D, Fidelis K, Tramontano A, Kryshtafovych A. New encouraging developments in contact prediction: Assessment of the CASP11 results. Proteins. 2016;84:131–44. doi:10.1002/prot.24943. 10.1002/prot.24943 PMC4834069 26474083

[33] McGuffin LJ, Bryson K, Jones DT (2000). The PSIPRED protein structure prediction server. Bioinformatics.

[34] Zhang Y, Skolnick J (2004). Scoring function for automated assessment of protein structure template quality. Proteins Struct Funct Bioinforma.

[35] Kim DEE, Dimaio F, Yu-Ruei Wang R, Song Y, Baker D (2014). One contact for every twelve residues allows robust and accurate topology-level protein structure modeling. Proteins Struct Funct Bioinforma.

[36] Sathyapriya R, Duarte JM, Stehr H, Filippis I, Lappe M. Defining an essence of structure determining residue contacts in proteins. Nussinov R, editor. PLoS Comput. Biol. Public Library of Science; 2009;5:e1000584. 10.1371/journal.pcbi.1000584 PMC2778133 19997489

[37] Cao R, Cheng J (2016). Protein single-model quality assessment by feature-based probability density functions. Sci Rep.

[38] Cao R, Bhattacharya D, Adhikari B, Li J, Cheng J. Large-scale model quality assessment for improving protein tertiary structure prediction. Bioinformatics. 2015; 31(12):i116-i123. http://bioinformatics.oxfordjournals.org/content/31/12/i116.short. 10.1093/bioinformatics/btv235 PMC4553833 26072473

[39] Cao R, Wang Z, Wang Y, Cheng J (2014). SMOQ: a tool for predicting the absolute residue-specific quality of a single protein model with support vector machines. BMC Bioinformatics.

[40] Bhattacharya D, Cheng J (2015). De novo protein conformational sampling using a probabilistic graphical model. Sci Rep.

[41] Bhattacharya D, Cao R, Cheng J (2016). UniCon3D: de novo protein structure prediction using united-residue conformational search via stepwise, probabilistic sampling. Bioinformatics.

[42] Jones DT (2001). Predicting novel protein folds by using FRAGFOLD. Proteins Struct Funct Genet.

[43] Pettersen EF, Goddard TD, Huang CC, Couch GS, Greenblatt DM, Meng EC (2004). UCSF Chimera - A visualization system for exploratory research and analysis. J Comput Chem.

